# Environmental factors shape the epiphytic bacterial communities of *Gracilariopsis lemaneiformis*

**DOI:** 10.1038/s41598-021-87977-3

**Published:** 2021-04-21

**Authors:** Pengbing Pei, Muhammad Aslam, Hong Du, Honghao Liang, Hui Wang, Xiaojuan Liu, Weizhou Chen

**Affiliations:** 1 Institute of Marine Sciences, Guangdong Provincial Key Laboratory of Marine Biotechnology and STU-UNIVPM Joint Algal Research Center, College of Science, Shantou University, Shantou, China; 2 Southern Marine Science and Engineering Guangdong Laboratory (Guangzhou), Guangzhou, China; 3 Faculty of Marine Sciences, LUAWMS, Lasbela, Pakistan

**Keywords:** Microbiology, Ecology, Environmental sciences, Planetary science

## Abstract

Macroalgae host various symbionts on their surface, which play a critical role in their growth and development processes. However, there is still incomplete understanding of this epiphytic bacteria-host algae interactions. This study comprehensively analysed variation of the epiphytic bacterial communities (EBC) composition of red macroalga *Gracilariopsis lemaneiformis* at different geographic locations and environmental factors (i.e., nitrogen and phosphorus), which shape the EBC composition of *G. lemaneiformis*. The composition and structure of EBC were characterized using high throughput sequencing of the V3-V4 hypervariable region of the 16S rRNA gene. The results revealed that epiphytic bacteria varied significantly among three different geographic locations in China, i.e., Nan’ao Island (NA), Lianjiang County (LJ), and Nanri Island (NR). Redundancy analysis (RDA) showed that the relative abundance of Bacteroidetes, Firmicutes, Verrucomicrobia, and Epsilonbacteraeota at NR were strongly positively correlated with total nitrogen (TN), total phosphorus (TP), nitrate nitrogen (NO_3_-N), and dissolved inorganic nitrogen (DIN), but negatively correlated with nitrite nitrogen (NO_2_-N). The relative abundance of Cyanobacteria at NA and LJ were strongly positively correlated with NO_2_-N, but negatively correlated with TN, TP, NO_3_-N, and DIN. Besides, the Mantel test results indicated that the EBC composition was significantly correlated with these environmental factors, which was also confirmed by Spearman correlation analysis. Thus, environmental factors such as NO_3_-N and DIN play a key role in the community composition of epiphytic bacteria on *G. lemaneiformis*. This study provides important baseline knowledge on the community composition of epiphytic bacteria on *G. lemaneiformis* and shows correlation between different epiphytic bacteria and their surrounding environmental factors.

## Introduction

Macroalgae are major habitat-forming organisms in marine ecosystems that play a critical role in building the physical structure of habitats^1,2^. They produce nutrients^3^ by releasing extracellular products into the surrounding environment during their growth process^4^, forming a unique microenvironment on the surface of the algal body^5^. Epiphytic bacteria release nutrient supplements^6^ such as vitamin B12^7^ and fatty acids^8^, regulate the growth of the macroalgae^4^, while some also produce antimicrobial compounds^9^. Besides, epiphytic bacteria regulate and prevent the settlement of other marine organisms. For example, the epiphytic bacteria of green alga *Ulva reticulata* produce bioactive compounds that inhibit the settlement of polychaete *Hydroides elegans*^10^ on macroalgal surfaces^8,11^.

Studies on biodiversity and community composition of epiphytic bacteria of macroalgae have been scientists’ focus in the last decade using culture dependent analysis. For instance, Burke et al.^12^ revealed that Alphaproteobacteria and Bacteroidetes were the dominant groups, while Rhodobacteriaceae, Sphingomonadaceae, Flavobacteriaceae, and Sapropiraceae were the dominant families of bacteria on the surface of green macroalgae collected from Bare Island, La Perouse. Similarly, Tujula et al.^13^ reported that Alphaproteobacteria and Bacteroidetes were the dominant epiphytic microbial communities on the surface of *Ulva australis* collected from Shark Point, Clovelly, NSW, Australia. Moreover, the epiphytic bacteria on *Ulva australis* were taxonomically and functionally distinct from the surrounding seawater^12,14^. These observations were confirmed by Roth-Schulze et al.^1,15^, who demonstrated^1^ that most of the OTUs abundant on *Ulva* surface are undetectable in the surrounding seawater. They also observed that the EBC of *Ulva* spp., are a result of planktonic bacterial communities selection, with these communities found to be both taxonomically and functionally distinct regardless of the host or surface-types^15^. Selvarajan et al.^16^ also found Proteobacteria, Bacteroidetes, Firmicutes, Cyanobacteria, Planctomycetes, Actinobacteria, and Verrucomicrobia as the dominant bacteria groups on the surfaces of eight different seaweeds collected from a rocky intertidal zone from the Indian Ocean at Cape Vidal, South Africa. Therefore, the authors concluded that the diversity of epiphytic bacteria isolated from different seaweeds is influenced mainly by secondary algal metabolites and elemental deposits on their surfaces, which activate genes that metabolize these substances in epiphytic bacteria^16^.

Recently, the epiphytic bacterial composition on macroalgae has been shown to be host-specific^15,17^ and regulated by environmental factors^17^. For instance, Aires et al.^17^ studied the spatial diversity of bacterial communities associated with two invasive *Asparagopsis* sp., i.e., *A. taxiformis* and *A. armata*, from north east of the Atlantic Ocean, and observed different EBC compositions of the same algal spp., isolated from two different locations i.e., continental and island habitats. Similarly, using a hierarchy design, Roth-Schulze et al.^15^ found unique taxonomic diversity to each host type, such as green, red, brown macroalgae, seagrass, rock, and seawater. The host-specificity of epiphytic microbial communities might be due to extracellular secondary metabolites secreted by the host during their life cycle. For example, chemical compounds detected on the surface of *Fucus vesiculosus*, were tested for bacterial settlement and community composition, and found to mediate colonization of epiphytic bacteria^18^. Nylund et al.^19^ also demonstrated that surface-bound antibacterial compounds produced by red alga *Bonnemaisonia asparagoides* had significant effect on the abundance and composition of EBC. While these studies indicate that the host-specificity of the EBC is closely related to the physicochemical properties of the host surface, few studies propose a lottery model for colonization of the algal surface, which attempts to explain the unusual lack of similarity in bacterial communities composition across different algae^12,14,20^. The lottery model, which was originally designed to explain the coexistence of reef fish species that occupy the same ecological niche^21,22^, seeks to explain the pattern of bacterial colonization on the surface of macroalgae^12^. This model suggested that hosts are colonized by ‘suitable’ bacteria from the surrounding species pool, resulting in high variability of bacterial communities structure across sites and among individual samples^12,20^.

Environmental factors such as temperature, salinity, light intensity, nitrogen, and biogeographic differences can also affect the composition of bacterial communities associated with macroalgae. For example, a 20%-50% increase in relative abundance of epiphytic bacteria from members of the *Rhodobacteraceae* family of *F. vesiculosus* was found with increasing temperature^23^. In addition to temperature, epiphytic bacteria associated with *F. vesiculosus* are influenced by salinity^24^, as demonstrated by Zhang et al.^25^, that the dominant bacterial communities associated with kelp were different under different salinity conditions. Similarly, Liao and Xu^26^ studied the correlation between EBC and nitrogen in *G. lemaneiformis* and found significant differences in the EBC with time and under different nitrogen concentrations, with significant differences found in the EBC at the apex and root of *G. lemaneiformis*. Interestingly, it has been observed that after transfering from the natural environment to an aquarium, macroalgae had different epiphytic microbial communities^27^ due to significant changes in microbial communities associated with *Delisea pulchra* for 15 days of aquarium maintenance.

It has previously been reported that the similarity in EBC composition of the same species decreases with increasing distance^28^. For example, minor similarity was found between the EBC of *U. australis* obtained from the two long distant positions (18,000 km apart) as compared to samples obtained from the two short distant places (500 m apart)^1^. Although the taxonomic composition of EBC of *Ulva* varied based on biogeographical locations, 70% of the detected core functions were independent of host species and biogeographic factors^1^.

**Figure 1:**
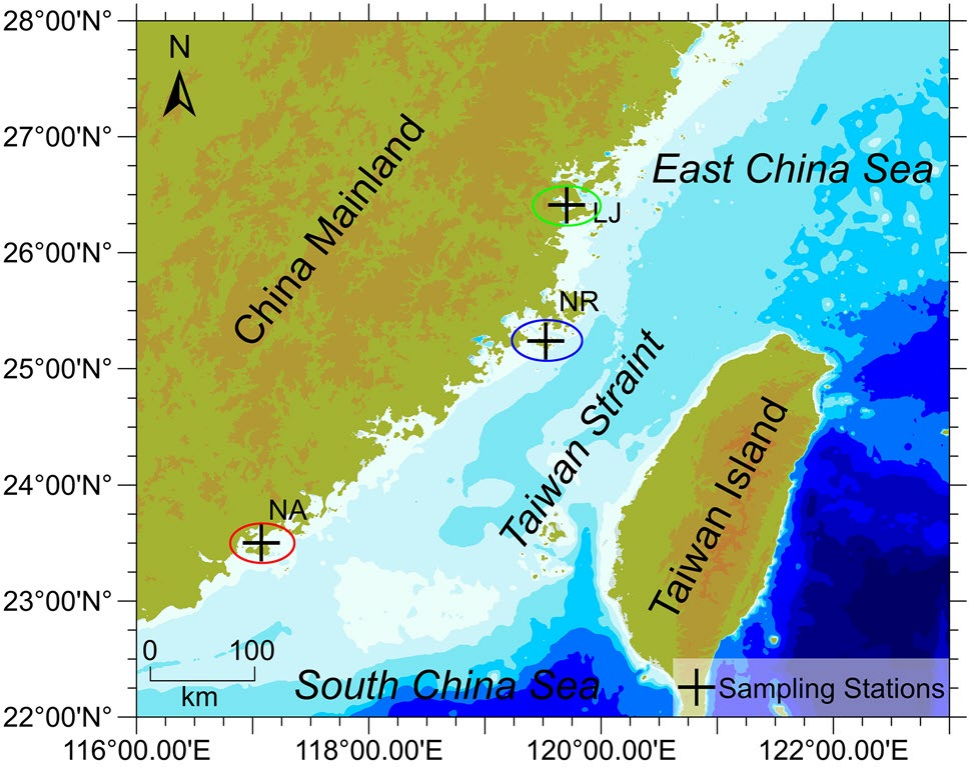
Sampling of *G. lemaneiformis* from three geographic locations. The red circle represents Nan’ao Island (NA), blue circle represents Nanri Island (NR), and green circle represents Lianjiang County (LJ).Surfer 14.0.599 (https://www.goldensoftware.com/) was used to create the map.

The red macroalgae *G. lemaneiformis* is not only important commercially as a food resourse, but also effective in decreasing eutrophication^29,30^. Thus, *G. lemaneiformis* has been cultivated on a large scale in China's southeast coastal areas, particularly Nan’ao Island, Nanri Island, and Lianjiang County, making it the third largest economic macroalgae after *Laminaria* and *Porphyra* ^31^. To the best of our knowledge, the diversity and community composition of epiphytic bacteria from *G. lemaneiformis* in large-scale artificial algal farms is still limited to a single geographic location^30^. Therefore, given the commercial importance of *G. lemaneiformis*, we undertook the first analysis of the EBC of *G. lemaneiformis* from three different geographic area in southeast China (Fig. 1) and examined the environmental factors that affect its composition.

## Results

### Physicochemical factors of the environment

**Figure 2:**
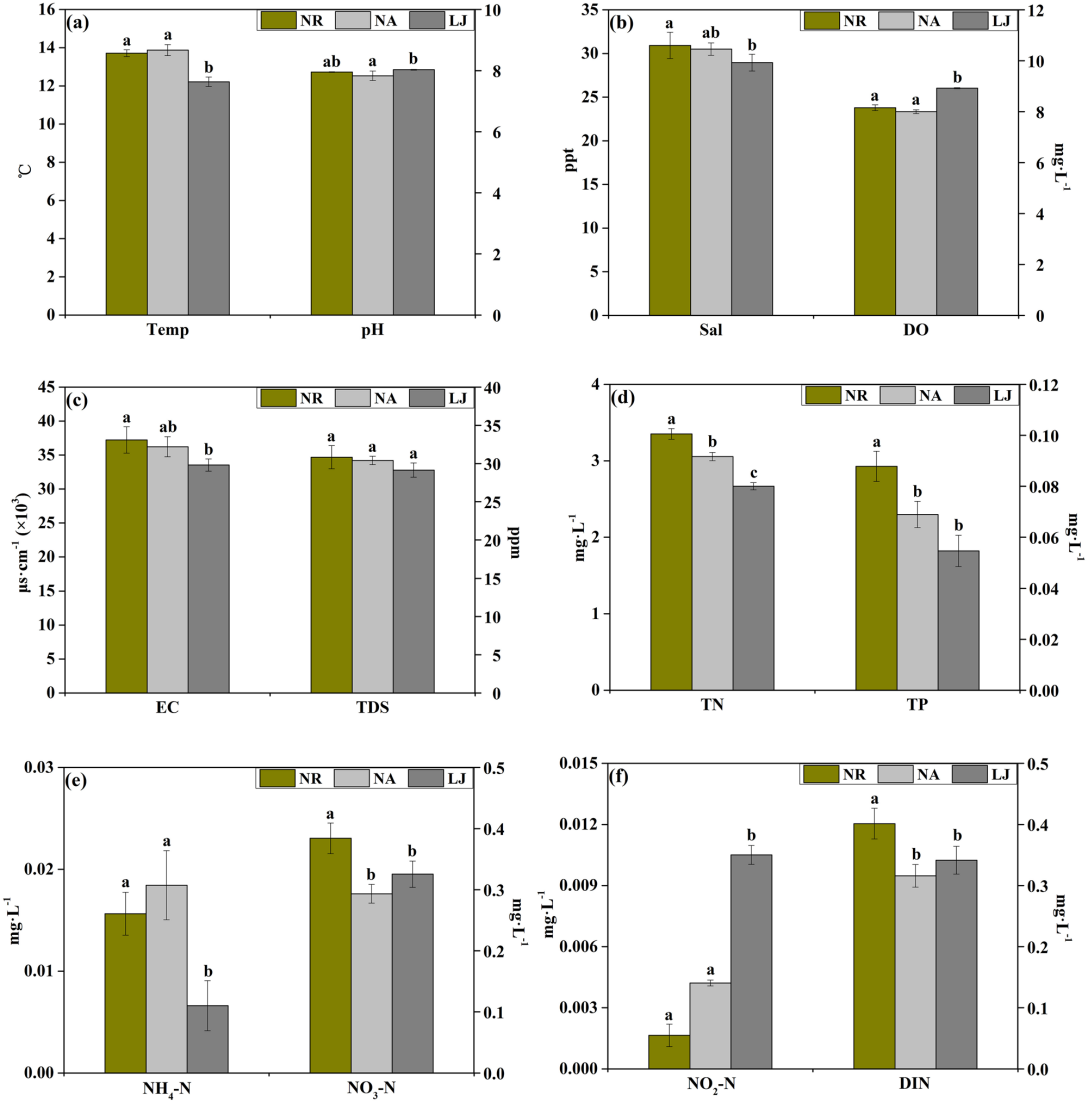
Average (± SD of nine replicates) values of seawater environmental factors surrounding *G. lemaneiformis* in the three locations. On each plot, y-axis on the left represents environmental factors on the left, while y-axis on the right corresponds to environmental factors on the right. Different letters (a, b, c) denote significant (*p* < 0.05) differences in seawater mean values between NR, NA, and LJ. *Temp* temperature, *Sal* salinity, *DO* dissolved oxygen, *EC* electrical conductance, *TDS* total dissolved solids, *TN* total nitrogen, *TP* total phosphorous, *NH*_*4*_*-N* ammonia, *NO*_*3*_*-N* nitrate, *NO*_*2*_*-N* nitrite, *DIN* dissolved inorganic nitrogen.

The physicochemical parameters of seawater from the study areas including temperature (Temp), pH, salinity (Sal), dissolved oxygen (DO), electrical conductance (EC), total dissolved solids (TDS), total nitrogen (TN), total phosphorus (TP), ammonia (NH_4_-N), nitrate (NO_3_-N), nitrite (NO_2_-N), and dissolved inorganic nitrogen (DIN) levels are shown in Fig. 2 & S. Tab 1. The temperatures in NR and NA were significantly higher (*p* < 0.05) than that in LJ (Fig. 2a), whereas DO concentration in NR and NA was significantly lower (*p* < 0.05) than that in LJ (Fig. 2b). There were no significant differences (*p* > 0.05) in pH between NR and NA, or between NR and LJ (Fig. 2a). The salinity and electrical conductance in NR were significantly higher (*p* < 0.05) than in LJ (Fig. 2b,c). There were no significant differences (*p* > 0.05) in salinity and electrical conductance between NR and NA, or between NA and LJ (Fig. 2b,c). Similarly, no significant differences in TDS were found among NR, NA, and LJ (Fig. 2c). The concentrations of TN and TP in NR were significantly higher (*p* < 0.05) than NA and LJ (Fig. 2d). On the other hand, there was no significant difference (*p* > 0.05) in NH_4_-N concentration between NR and NA, but was significantly higher (*p* < 0.05) than LJ (Fig. 2e). The levels of NO_3_-N and DIN in NR were significantly higher (*p* < 0.05) than NA and LJ (Fig. 2e,f), whereas the concentration of NO_2_-N in NR was significantly lower (*p* < 0.05) than LJ but not (*p* > 0.05) NA (Fig. 2f).

### Diversity of epiphytic bacterial communities on *G. lemaneiformis*

**Figure 3:**
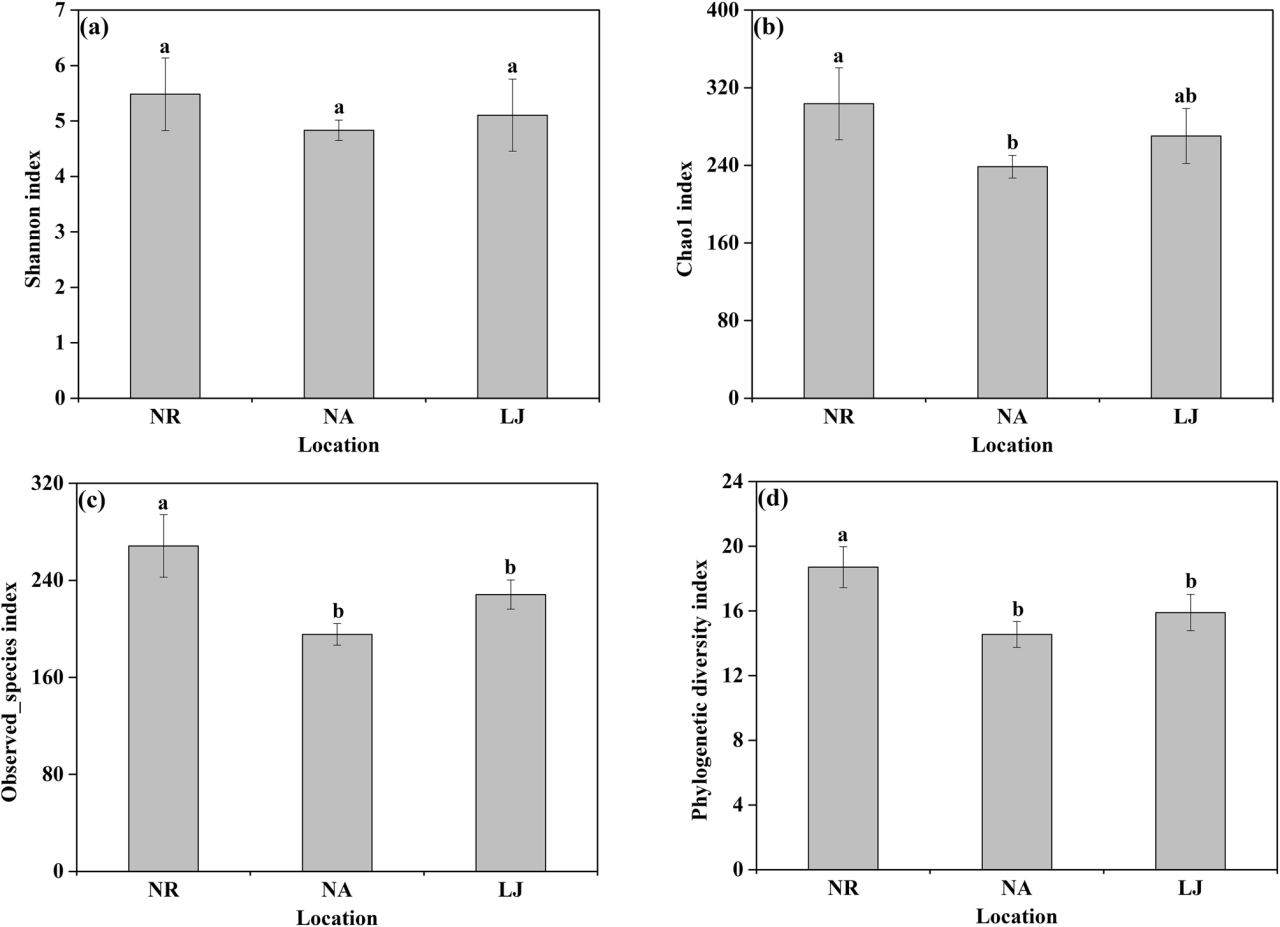
Shannon index **(a)**, Chao1 index **(b)**, Observed_species index **(c)** and Phylogenetic diversity index **(d)** showed the α-diversity of EBC on *G. lemaneiformis* at NR, NA, and LJ. Different letters (a, b) denote significant (*p* < 0.05) differences in mean value between NR, NA, and LJ in *G. lemaneiformis* samples.

Twenty-three algal samples (i.e., NR samples: 2 NR1, 3 NR2, 3 NR3; NA samples: 3 NA2, 3 NA3; LJ samples: 3 LJ1, 3 LJ2, 3 LJ3) were analyzed using Hiseq sequencing of the 16S rRNA gene. A total of 1,234,507 effective sequences were obtained with an average of 45,722 reads per sample (n = 23). Sequences were clustered into operational taxonomic units (OTUs) at the 97% similarity level to generate 6,238 OTUs from the 23 samples. The OTUs were classified into 11 phyla, 21 classes, 52 orders, 94 families, and 196 genera. The Shannon index of the EBC at NR was 5.48 ± 0.65 per sample, which was higher than that at NA (4.83 ± 0.18) and LJ (5.10 ± 0.65) (Fig. 3a and S. Tab. 2). The Chao1 index of the EBC at NR was significantly higher (*p* < 0.05) than that at NA and LJ (Fig. 3b and S. Tab. 2). Besides, the observed species index was the highest (*p* < 0.05) in the EBC at NR compared with NA and LJ (Fig. 3c and S. Tab. 2). Similarly, the EBC at NR had the highest (*p* < 0.05) phylogenetic diversity (PD_whole_tree index) compared with NA and LJ (Fig. 3d and S. Tab. 2).

### Geographic comparison of EBC on the surface of *G. lemaneiformis*

**Figure 4:**
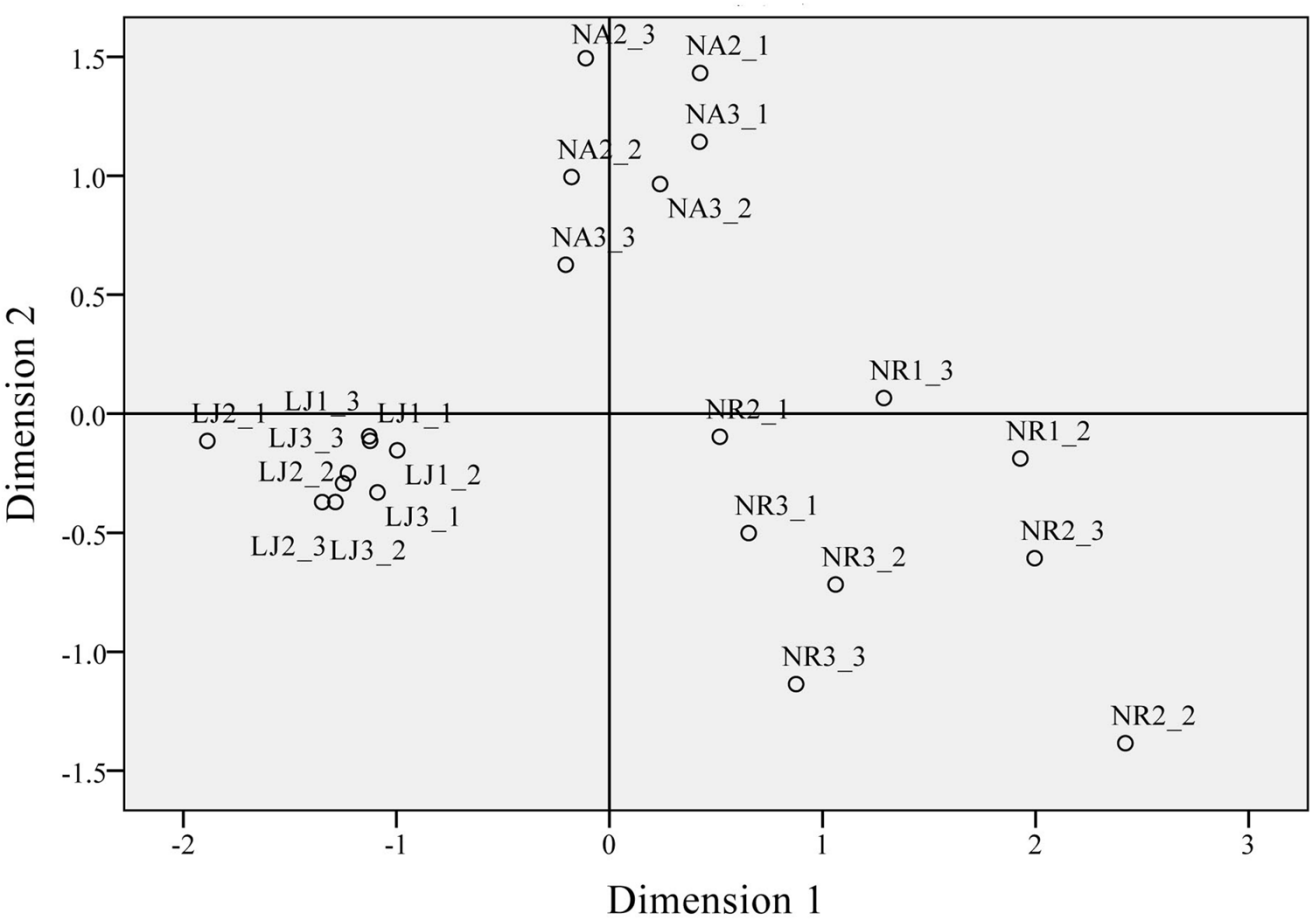
Non-metric multidimensional scaling (nMDS) based on Bray–Curtis measure. nMDS shows the differences and similarities of EBC on *G. lemaneiformis* at NR, NA and LJ.

Based on the analysis of OTUs, an NMD (Non-metric multidimensional) ordination biplot indicated clear clustering of EBC. The stress and RSQ (Squared correlation) values were 0.14391 and 0.91376, respectively (Fig. 4). The *G. lemaneiformis* samples at LJ generally clustered together, with a similar phenomenon also observed at NA and NR. Notably, the composition of EBC at NA and LJ were more similar compared with that at NR based on Dimension 1.

**Figure 5:**
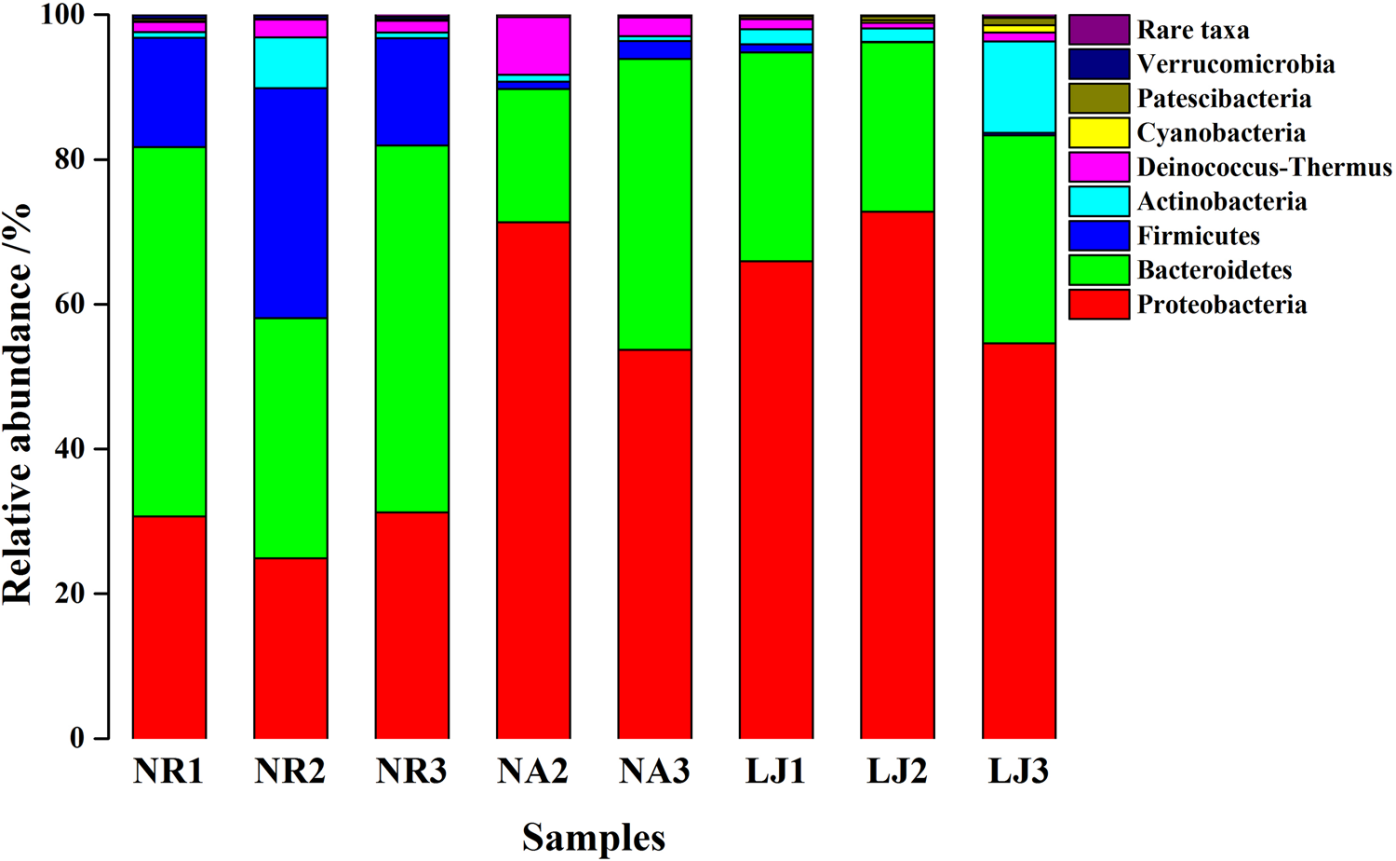
The EBC composition at phylum level on *G. lemaneiformis* at NR, NA, and LJ. Species richness represented less than 0.5% of the total bacteria in all samples were grouped into Rare taxa.

The EBC on the surface of *G. lemaneiformis* were considerably different in the three geographic locations (Fig. 5). Bacteroidetes was the most predominant phylum on the surface of *G. lemaneiformis* in NR, with a relative abundance of 44.982%, followed by Proteobacteria (28.941%) and Firmicutes (20.565%). Samples (*G. lemaneiformis*) collected from NR exhibited significantly (*p* < 0.05) diverse EBC on their surfaces compare with samples from NA and LJ. For examples, Proteobacteria was the most predominant phylum on the surface of *G. lemaneiformis* in NA and LJ, with relative abundances of 61.727% and 64.437%, respectively, which was significantly higher than NR (*p* < 0.05). The second dominant phylum was Bacteroidetes, with relative abundances of 30.140% at NA and 27.008% at LJ, which were significantly lower than that in NR (*p* < 0.05). On the other hand, the relative abundance of Firmicutes in NR was significantly higher than NA and LJ (*p* < 0.05), while the abundance of Deinococcus-Thermus in NA was significantly higher than NR and LJ (*p* < 0.05).

### Redundancy analysis and heat map

**Figure 6:**
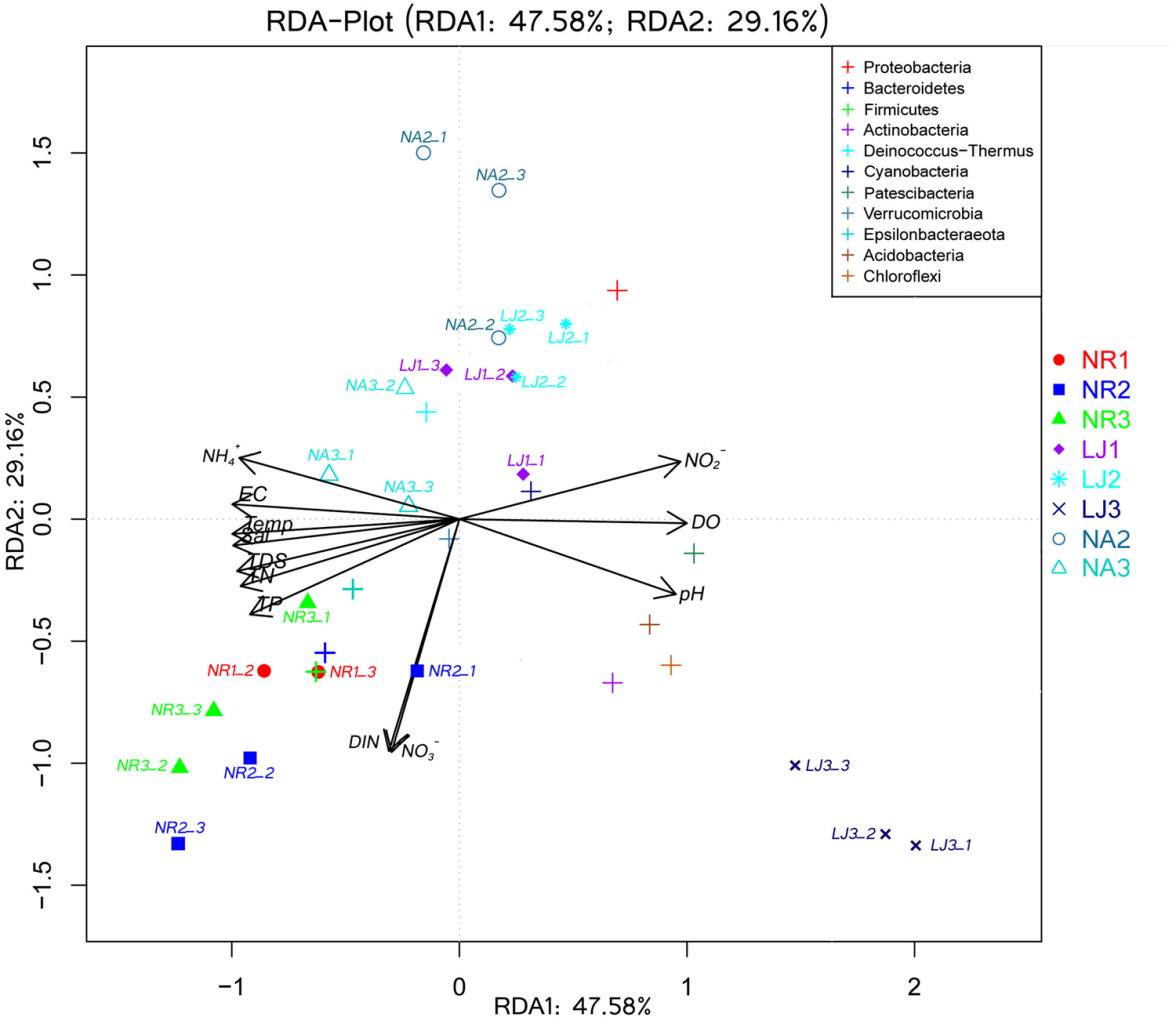
Redundancy analysis (RDA) of phyla (different color plus sign) in samples of epiphytic bacteria on *G. lemaneiformis* and environmental factors (black arrows) at NR, NA, and LJ.

**Figure 7:**
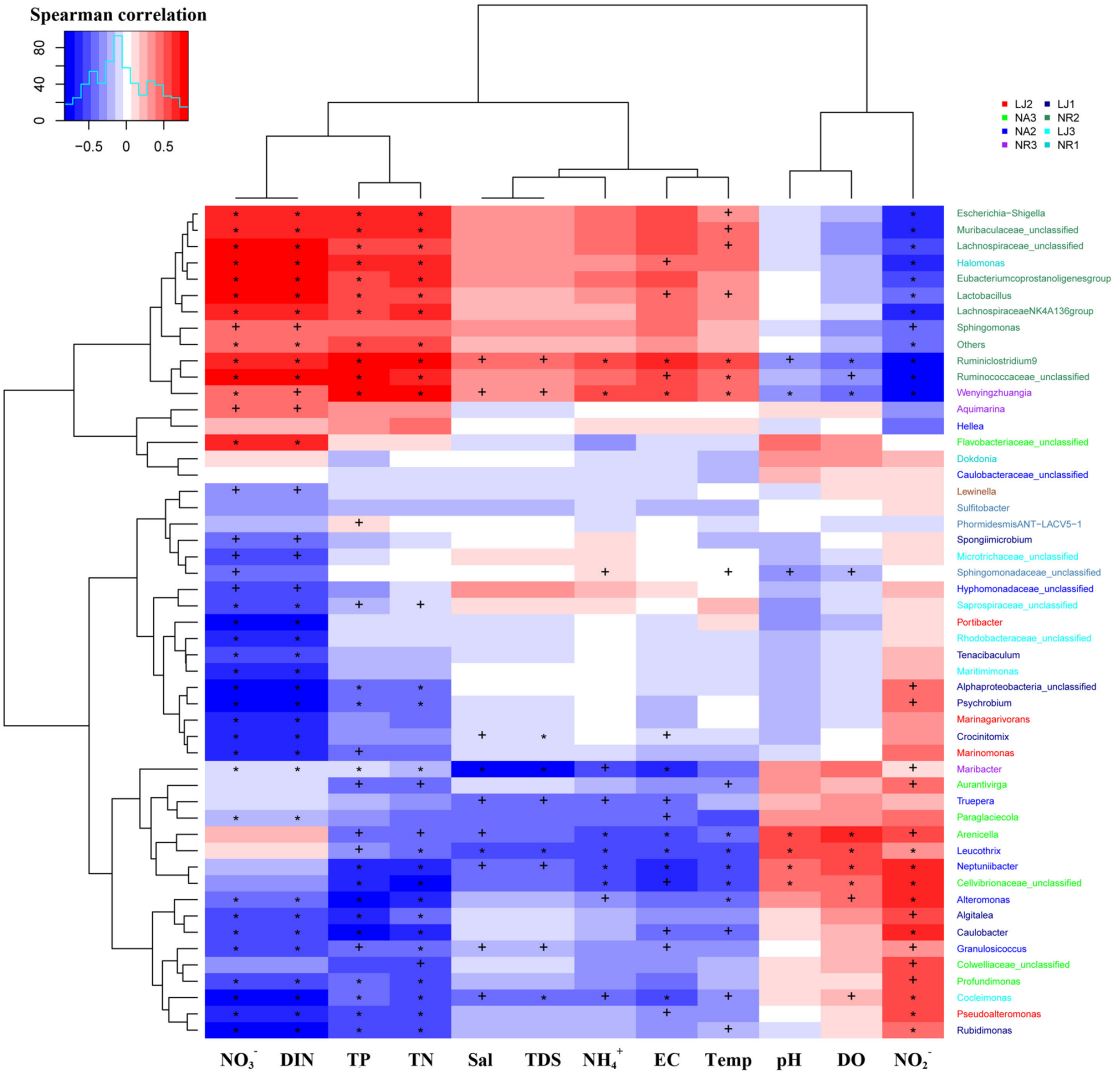
Heat maps showing correlation between the top 50 bacterial genera in species abundance of epiphytic bacteria on *G. lemaneiformis* and environmental factors in seawater. X and Y cladogram depict environmental factors and species clustering tree, respectively. Values of Spearman correlation coefficients are color coded in the heat map legends. The species on the right of figure are color coded from different samples. ‘ + ’ represents significant differences (*p* < 0.05), ‘*’ represents highly significant differences (*p* < 0.01). *Temp* temperature, *Sal* salinity, *DO* dissolved oxygen, *EC* electrical conductance, *TDS* total dissolved solids, *TN* total nitrogen, *TP* total phosphorous, *NH*_*4*_*-N* ammonia, *NO*_*3*_*-N* nitrate, *NO*_*2*_*-N* nitrite, *DIN* dissolved inorganic nitrogen. R (version 3.5.1; http://www.r-project.org/) software was used to create the figure. The ‘Corr. test’ function of psych package in R was used for making this figure.

Redundancy analysis (RDA) was used to explore the correlations between epiphytic bacteria on *G. lemaneiformis* at the phylum level and environmental factors (Fig. 6). The RDA results showed that epiphytic bacteria at NR, NA, and LJ clustered in groups. RDA1 and RDA2 explained 47.58% and 29.16% of the total variance, respectively. At NR, NO_3_-N, DIN, TN, and TP were strongly positively correlated with the relative abundance of Bacteroidetes, Firmicutes, Verrucomicrobia, and Epsilonbacteraeota, whereas NO_2_-N was negatively correlated with the EBC. Similarly, NO_2_-N was strongly positively correlated with the relative abundance of Cyanobacteria, whereas NO_3_-N, DIN, TN and TP showed negative correlations with the relative abundance of Cyanobacteria at NA and LJ. The RDA results together with Pearson correlation analysis (Fig. 7) revealed that NO_3_-N and DIN had a significant influence (*p* < 0.05) on macroalgal and microorganisms distribution. When the Mantel tests was used to examine the contribution of environmental factors to the assembly of community composition of epiphytic bacteria on *G. lemaneiformis*, the results (at both phylum and genus level) showed that community composition correlated significantly with NO_3_-N, NO_2_-N, DIN, TN, and TP (Table 1). There was also significant correlations between temperature, pH, DO and the genus communities composition.

**Table 1:** Mantel correlations showing the relationship between environmental factors and communities composition at phylum and genus level. Bold-faced entries indicate significance at *p* < 0.01.

Environmental factors	Phylum		Genus	
	Mantel score (r)	P-value	Mantel score (r)	P-value
Temp (°C)	0.071	0.172	**0.325**	**0.001**
pH	0.036	0.308	**0.388**	**0.001**
Sal (ppt)	0.046	0.291	0.093	0.133
DO (mg·L^−1^)	0.065	0.173	**0.372**	**0.001**
EC (μs·cm^−1^)	0.037	0.327	0.095	0.155
TDS (ppm)	0.038	0.327	0.074	0.153
NH_4_-N (mg·L^−1^)	– 0.097	0.227	0.051	0.271
NO_3_-N (mg·L^−1^)	**0.352**	**0.002**	**0.299**	**0.002**
NO_2_-N (mg·L^−1^)	**0.625**	**0.001**	**0.732**	**0.001**
DIN (mg·L^−1^)	**0.308**	**0.007**	**0.249**	**0.008**
TN (mg·L^−1^)	**0.330**	**0.004**	**0.445**	**0.001**
TP (mg·L^−1^)	**0.395**	**0.002**	**0.460**	**0.001**

Spearman correlation analysis was used to investigate how environmental factors affect the EBC compositions on *G. lemaneiformis*. The results revealed a relation between the composition of EBC on *G. lemaneiformis* and environmental factors of the surrounding seawater, as shown by the heat map based on Spearman correlations (Fig. 7). The variation of EBC at the genus level showed that all epiphytic bacterial composition, except *Dokdonia* and *Maribacter* at NR, exhibited a positive correlation with NO_3_-N, DIN, TN, and TP (0.31 < *r* < 0.81), but a negative correlation with NO_2_-N (-0.77 < *r* < -0.35). Notably, the abundance of *Escherichia-Shigella* in samples at NR2 was significantly positively correlated with NO_3_-N, DIN, TN, and TP. The abundance of all epiphytic bacterial composition except *Flavobacteriaceae*_unclassified, *Hellea*, *Arenicella*, *Leucothrix,* and *Caulobacteraceae*_unclassified at NA showed positive correlation with NO_2_-N (0.10 < *r* < 0.70), but negative correlation with NO_3_-N, DIN, TN, and TP (-0.77 < *r* < -0.11). Similarly, the abundance of all epiphytic bacterial compositions except *Sphingomonadaceae*_unclassified and *Phormidesmis_ANT-LACV5-1* at LJ correlated positively with NO_2_-N (0.06 < *r* < 0.70), but negatively with NO_3_-N, DIN, TN, and TP (-0.82 < *r* < -0.05).

### Functional prediction and comparative analysis

**Figure 8:**
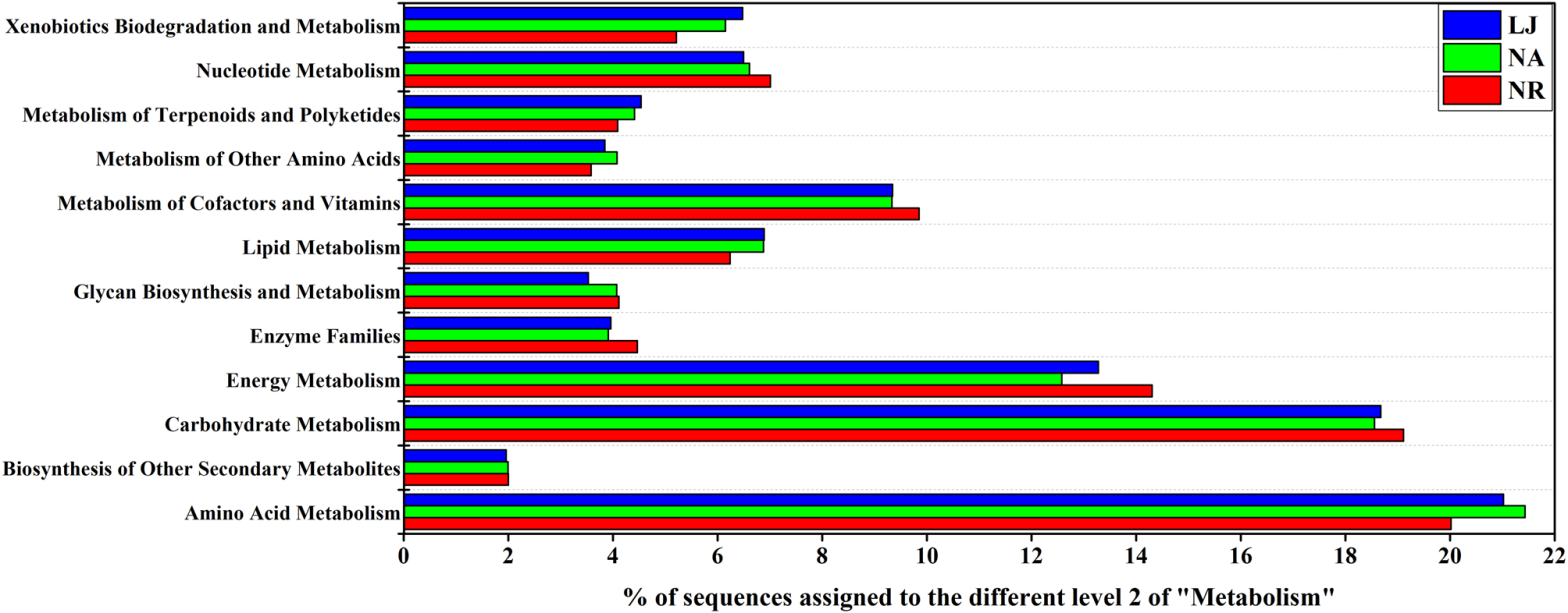
Percentage of metagenomic sequences of functional composition of Level 2—metabolism—of the EBC on *G. lemaneiformis* at NR, NA, and LJ.

To determine the functional profile of the epiphytic bacteria communities on *G. lemaneiformis* from the three geographic locations, phylogenetic investigation by reconstruction of unobserved states (PICRUSt) was used to analyze and predict the functional capabilities of EBC on *G. lemaneiformis*. The KEGG pathway analysis from all the predicted metagenomes revealed that metabolism was the most enriched functional composition of level 1 in all groups i.e., NR: 50.060%, NA: 51.384%, and LJ: 52.717%, respectively (S. Figure 3). Within the metabolism assignments for all groups, the enrichment of amino acid, carbohydrate, and energy metabolisms were the highest (Fig. 8 and S. Tab. 4). The metabolisms of amino acid showed ‘amino acid related enzymes’, while ‘arginine and proline’ were found as most and ‘phenylalanine’ as least predominant in all groups (S. Figure 4). ‘Amino sugar, nucleotide sugar’, ‘glycolysis/gluconeogenesis’, ‘pyruvate’ in carbohydrate and ‘oxidative phosphorylation’ except ‘photosynthesis-antenna proteins’ in energy metabolisms were found as the most abundant in all groups (S. Figures 5 and 6).

## Discussion

To the best of our knowledge, this is the first study that used next generation sequencing to evaluate geographic and environmental specificity of epiphytic bacterial communities (EBC) on cultivated red macroalgae *G. lemaneiformis*. The focus of previous studies have been on spatial and temporal diversity of EBC on macroalgae^13,17,20^, while few reports have attributed differences in the EBC composition on macroalgae surface from different geographic locations to environmental selections^17,32^. Although environmental selection can produce substantial biogeographic patterns in the global microbe population^33^, there are selective mechanisms that determine the assemblage of species in one environment or the other^12^. Thus, to better understand this phenomenon, we investigated the EBC composition, their correlation at the spatial level, and the role of different environmental factors (e.g., nitrogen and phosphorous) in shaping EBC composition. We demosndtrated that the similarity among EBC was higher in *G. lemaneiformis* from sampling sites that are 300 m apart. For instance, at NR 17.5%-66.92% similarity was found, with 12.5%-64.77% similarity at NA, and 50%-75.07% similarity at LJ, when compared with *G. lemaneiformis* samples from NA and LJ which are 500 km apart, and exhibit only 7.5% similarity. The similarity of the EBC at different sampling sites in any of the three locations was much higher than that between the two most distant locations (NA and LJ, 500 km apart). This observation fits into the distance-decay of similarity model, where decrease in the similarity of microbial communities is related to an increase in geographic distance^28^. Our findings are consistent with the study by Roth-Schulze et al.^1^, where variations in the similarity of EBC correlated with distance in marine, soil, sediment, and plant-associated ecosystems^1^.

Our study revealed considerable variations of EBC composition on *G. lemaneiformis* among the three geographic locations (NR, NA, and LJ), where the EBC were mainly composed of *Flavobacteriaceae, Saprospiraceae, Muribaculaceae* (Bacteroidetes), *Hyphomonadaceae, Sphingomonadaceae, Rhodobacteraceae* (Alphaproteobacteria), *Cellvibrionaceae, Thiohalorhabdaceae, Nitrincolaceae, Thiotrichaceae, Halomonadaceae* (Grammaproteobacteria), *Ruminococcaceae, Lachnospiraceae* (Firmicutes), *Microtrichaceae* (Actinobacteria), *Trueperaceae* (Deinococcus-Thermus) and Rare taxa (S. Figure 1 and S. Tab. 3). These observations are consistent with previous studies of marine algal-associated bacterial communities. For example, the EBC on the surface of the red algae, *Delisea pulchra*, comprised of *Rhodobacteraceae, Sphingomonadaceae, Flavobacteriaceae, Planctomycetaceae,* and unclassified Grammaproteobacteria^34^, whereas the green alga *U. australis* hosts Alphaproteobacteria, Bacteroidetes, Planctomycetes, and unclassified Grammaproteobacteria^12^. *Fucus vesiculosus* is reported to be associated with high proportions of Alphaproteobacteria, Bacteroidetes, Verrucomicrobia, and Cyanobacteria in summer, but mainly host Grammaproteobacteria in winter^35^. Low abundance of epiphytic bacteria in the phyla Cyanobacteria and Verrucomicrobia were found in this study, which have also previously been reported to be widespread in different seaweeds^16,35^, suggesting that members of these phyla play an important role in algae-bacteria interaction. Cyanobacteria are widely dispersed in rivers, lakes, and the ocean, with the ability to withstand harsh environmental conditions^36^. The core microbial communities are often defined as the suite of members shared among microbial communities from similar habitats^37^. Thus, discovering the core microbial communities is important for understanding stable and consistent components across complex microbial assemblages. In this study, significant differences in the core EBC were found across the three geographic locations (S. Figure 2). For instance, Cyclobacteriaceae, Pseudomonadaceae, Sphingobacteriaceae, Xanthobacteraceae, Burkholderiaceae, Beijerinckiaceae, Nocardiaceae, Rhizobiaceae, and Micrococcaceae were the dominant bacteria family on *G. lemaneiformis* at NR, while at NA and LJ, Veillonellaceae, Bifidobacteriaceae, Coriobacteriaceae, Porticoccaceae, Bacillaceae and Sandaracinaceae, Staphylococcaceae, Corynebacteriaceae, Blastocatellaceae, Rhizobiales, Flammeovirgaceae, Shewanellaceae were dominant.

Epiphytic bacteria on the surface of different macroalgae are vital and beneficial to the hosts. For example, cross-kingdom chemical signals derived from members of Halomonas (*Halomonadaceae*), Roseobacter (*Rhodobacteraceae*), and Sulfitobacter (*Flavobacteriaceae*) are essential for the thallus development of *Ulva mutabilis* (Chlorophyta)^38^. Although few studies have revealed that dimethylsulfoniopropionate (DMSP) play a key role in macroalgal-bacterial interactions^39^, Alphaproteobacteria, which are morphologically and metabolically diverse^13^, have a critical role in the assimilation of DMSP in oceans and contribute significantly to global sulphur cycling^40^. While DMSP produced by macroalgae including *Ulva* sp. that usually attract some bacteria^39^, some members of the family *Hyphomonadaceae* are widely dispersed in marine environment and play a vital role in mineralizing dissolved organic matters in oligotrophic waters^17^. Based on our findings, it seems the ability of *G. lemaneiformis* to grow in the three different oligotrophic waters may partly benefit from the mineralization by these microorganisms on its surface. After all, a previous study by Holmström et al.^41,42^ had shown that many bioactive compounds produced by the genus *Pseudoalteromonas* presented on the surface of *Ulva lactuca* play an important role in chemical defense against biofouling in marine environment.

Several studies on holobionts have shown host-specificity of EBC within different species or geographic differences of EBC between different locations^1,15,17,20,32^. For instance, the EBC on different host species were taxonomically and functionally distinct, which is not due to the phylogeny of the host but to the physicochemical properties of the host^15^. Thus, Lachnit et al.^43^ have suggested that the difference in the EBC on different algal hosts are due to the physiochemical properties of the macroalgae surface, which allows the settlement and colonization by specific bacteria. In terms of geographic diversity, Roth-Schulze et al.^1^ demonstrated that the same algal-genus across different regions can harbor different microbial communities at the taxonomic and functional level, due to local geographical conditions and host specificity.

When the specificity of *G. lemaneiformis* associated EBC and their correlation with different environmental factors was examined, the concentrations of TN, TP, NO_3_-N, and DIN at NR were found to be significantly higher compared to NA and LJ (*p* < 0.05), while NO_2_-N concentration at NR was significantly lower compared to LJ (*p* < 0.05) and NA (*p* > 0.05), although all sites had similar environmental conditions i.e., temperature, pH, salinity, dissolved oxygen, electrical conductance, and total dissolved solids. Changes in environmental conditions, such as nutrient concentration, nutrient ratio, and temperature, can affect the physicochemical properties of macroalgae^44^. For instance, Van Alstyne^45^ reported that *Ulva lactuca* and *Ulva obscura* grown in high nitrogen concentration have higher DMSP content than in low nitrogen concentration, suggesting that different environmental conditions can affect the content of algal-associated compounds. Increasing evidence shows that compounds associated with algal surface can mediate epiphytic bacterial colonization, abundance, and community composition of macroalgae^18,19,46^. Given that these studies suggest that the differences in EBC composition of macroalgae due to changes in environmental factors can be attributed to variations in physicochemical properties on macroalgae surface, we speculate that the variation of the EBC on *G. lemaneiformis* was probably due to differences in algal-associated compounds caused by environmental conditions. Generally, environmental factors that affect the diversity, composition, and structure of microbial communities include temperature, pH, salinity, and DO^23,24,30^. Since many marine microbes require oxygen for growth and survival, DO is crucial to microbial communities in coastal ecosystems^30^. Thus, a higher DO concentration could relate to an increased taxonomic and functional diversity of microbial communities. In our current study, the concentration of DO at NR and NA was significantly lower (*p* < 0.05) compared to LJ, although the EBC diversity in NR was higher than that in NA and LJ. These results suggest that DO might not be the main factor that affects the diversity of EBC on *G. lemaneiformis*. Even though temperature is one of the important environmental factors that affect microbial communities in many habitats^23^, no significant differences in ocean water temperature was observed among the three sampling sites (NR, NA, and LJ). This indicates that temperature is not the main factor that affects the diversity of EBC on *G. lemaneiformis*, which is consistent with the report by Zozaya-Valdés et al.^27^ that environmental factors other than temperature seems to impact on alga-associated microbiome.

Considerable changes in EBC on the surface of *G. lemaneiformis* was found at the three geographic locations, regardless of the taxonomic level (Fig. 5, S. Figure 1 and S. Tab. 3). At phyla level (Fig. 5), Bacteroidetes was the most predominant phylum at NR, whereas Proteobacteria was dominant at NA and LJ. Our results are similar to a previous study on surface-associated bacterial communities on macroalgae, which revealed that epiphytic bacteria of *U. australis* are dominated by Proteobacteria and Bacteroidetes^12^. Although at the family level (S. Tab. 4), the relative abundance of *Muribaculaceae*, *Ruminococcaceae,* and *Lachnospiraceae* at NR was significantly higher (*p* < 0.05) than NA and LJ, no significant differences (*p* > 0.05) were observed between NA and LJ. This indicates that there are significant differences in the EBC composition on *G. lemaneiformis* between NR and NA and between NR and LJ, but not between NA and LJ, which is contrary to previous reports that algal EBC varies between different locations^1,32^. Probably the explanation for this observation could be due to secretions of some compounds on the surface of *G. lemaneiformis* that regulates EBC composition.

When RDA^47^ and Spearman correlation analysis^30^ were employed, the EBC on *G. lemaneiformis* at NR exhibited positive correlation with TN, TP, NO_3_-N, and DIN, and negative correlation with NO_2_-N (Figs. 6 and 7). A clinically important bacterial genus, *Escherichia-Shigella*, was found on the *G. lemaneiformis* at NR2, and was significantly correlated with NO_3_-N, DIN, TP, and TN (Fig. 7). *Escherichia-Shigella* is an enteric pathogen that seeps from waste water treatment plants^48^, and can secrete toxins to the surrounding environment^49^. The correlation between *Escherichia-Shigella* abundance and environmental factors could be of ecological or health concerns^50^. The EBC on *G. lemaneiformis* at NA and LJ correlated positively with NO_2_-N and negatively with TN, TP, NO_3_-N, and DIN. Thus, the differences in *G. lemaneiformis* associated EBC from the different geographic locations is due to environmental factors but not geographical locations. These findings are consistent with a previous study in which *Asparagopsis*-associated bacterial communities were observed to be modulated by environmental conditions^17^. This is further supported by Roth-Schulze et al.^1^, who reported that most *Ulva*-associated bacterial communities are horizontally derived from the environment because macroalgae *U. australis* isolated from distinct geographical locations are found to share only two low-abundance OTUs. However, in the current study, *G. lemaneiformis* from NA and LJ (which are 500 km apart) only shared 7.5% similarity. Intriguingly, environmental factors but not geographical locations account for the differences in the EBC on *G. lemaneiformis*, which is contrary to the previous report by Roth-Schulze et al.^1^. Therefore, we assume that the EBC on *G. lemaneiformis* is regulated by environmental factors such as nitrogen and phosphorus rather than geographical locations.

Interestingly, replicate samples of *Gracilaria* species from the same location showed some variability in their EBC composition, as indicated by Bray–Curtis similarity of 17.5–66.92% at NR, 12.5–64.77% at NA, and 50–75.07% at LJ. This observation is similar to previous studies, where a high level of intraspecies variability of EBC was found associated with macroalgae^1,14^. The depth of sequencing^13^, or the lottery model in which random variation can be seen in the recruitment of the EBC on algal surface^21^ might account for these observations. The lottery model proposes that recruitment of species with the same tropic abilities in any ecosystem follows a stochastic fashion i.e., who ever gets there first wins the space, but they must share similar ecologies^21,51,52^. Thus, to further ascertain that environmental factors indeed play a role in the selection of the EBC on macroalgae, future studies would explore comparative EBC composition of other algae in the presence of similar environmental factors.

Although considerable differences in the composition of EBC on *G. lemaneiformis* was observed at the three geographic locations, the functional composition of EBC were similar. There is an emerging consensus that the bacterial community composition on macroalgae is mainly driven by functional genes rather than taxonomic composition^14,16^. In the current study, the functional capabilities of EBC on *G. lemaneiformis* at the different locations were similar, which indicated similar functions of these bacterial communities at these locations. The fundamental factor responsible for the differences in the composition of bacterial communities was the microenvironment, which is established by the physiological and biochemical properties of the algal host^3,16^. We found bacterial genes associated with the amino acids glycine, alanine, arginine, proline, glutamic, and aspartic acids^53^ (S. Figure 4). Given that *G. lemaneiformis* is rich in proteins and a source of phycoerythrin production ^54^, algal proteins can be selectively used by the bacteria on the surface of *G. lemaneiformis*, which might explain the higher percentage of bacterial genes assigned to amino acid metabolism (Fig. 8). The presence of the amino acid metabolism associated genes indicates an adaptive mechanism of epiphytic bacteria to use organic matter from macroalgae for growth. Besides, the abundant functional genes related to carbohydrate metabolism suggest an involvement in the mineralization of dissolved organic matter under the oligotrophic environment of coastal water (Fig. 8). Similar findings have previously been reported by Selvarajan et al.^16^, where a higher abundance of bacterial functional genes associated with carbohydrate metabolism were found in all seaweeds at intertidal zones of Mission Rocks, Cape Vidal, Leven Point, South Africa. Members of the Hyphomonadaceae family are reported to be the main contributors to mineralization of dissolved organic matter in oligotrophic conditions^17^. In the current study, Hyphomonadaceae were also found to be highly abundant in the EBC on *G. lemaneiformis* (S. Tab. 3). Thus, we speculate that Hyphomonadaceae harbor significant number of functional genes related to carbohydrate metabolism, which might be the reason for pyruvate metabolism being the most abundant in all groups (S. Figure 5), because energy from pyruvate decomposition is stored in the form of high-energy phosphates for microbial growth and reproduction^55^. From an ecological perspective, macroalgae may also exploit QS inhibitors and antimicrobial compounds produced by numerous epiphytic bacteria to protect their surfaces from pathogens, herbivores and fouling organisms^56^. Although functional genes associated with the biosynthesis of other secondary metabolites showed the lowest abundance in ‘Metabolism’ (Fig. 8), these functions cannot be ignored in the chemical defense process of algae. The limitation of the current study is that all the inferences are based on predicted functions by PICRUSt annotation, which cannot completely substitute metagenomic research, hence, there would be inherent inaccuracies in interpreting functional biogeography in some ecosystems.

## Conclusion

The current study demonstrates that EBC associated with *G. lemaneiformis* varied significantly at three geographic locations. The relative abundance of EBC at NR was strongly positively correlated with total nitrogen, total phosphorus, nitrate, and dissolved inorganic nitrogen, whereas the EBC at NA and LJ only strongly correlated positively with nitrite. Therefore, environmental factors such as nitrate and dissolved inorganic nitrogen are the key factors that shape the EBC composition on *G. lemaneiformis* but not geographic locations. This is the first study that provides insight into the EBC composition on *G. lemaneiformis* at three geographic locations and shows that environmental factors influence their composition.

## Materials and methods

### Collection location and sampling

Individual thalli from *G. lemaneiformis* were collected from three different locations, Nan’ao Island of Guangdong Province (NA, 117°6′40″E, 23°29′9″N), Nanri Island of Fujian Province (NR, 119°33′13″E, 25°13′29″N) and Lianjiang County of Fujian Province (LJ, 119°43′35″E, 26°23′31″N) between January and February 2018. The topographic data for the map in this study was obtained from the General Bathymetric Chart of the Oceans (GEBCO, https://www.gebco.net/). The latitude and longitude were measured by GPS (Garmin GPS72H) from the sampling locations. The map was created by Surfer 14.0.599 (https://www.goldensoftware.com/). The selection of sampling seasons was based on the vigorous growth stage of *G. lemaneiformis* during this period. At each location, three sampling sites (ca. 300 m apart) were randomly selected. Three replicate surface (0.5 m depth) water were collected at each sampling site (1.0 L) for physicochemical analyses. At NA, three replicate *G. lemaneiformis* were collected at two sampling sites except for NA1 (due to inclement weather). At NR and LJ, three replicate *G. lemaneiformis* were collected from each sampling site. The NR1.1 sample was missing due to an experimental error. Full details of the sampling information can be found in S. Tab. 5. All algal samples were stored in sterile polyethylene bags along with seawater. Both algal and seawater samples were transported to the laboratory under cold conditions.

### Determination of physicochemical factors

The seawater temperature, salinity, pH, dissolved oxygen (DO), electrical conductance (EC), and total dissolved solids (TDS) were measured using In-Situ SMARTPOLL MP (U.S.A). Briefly, seawater samples were filtered with a 0.45 μm mixed cellulose ester microporous filter membrane (MF-Millipore HAWP04700, USA) within 2 h. Ammonia (NH_4_-N), nitrate (NO_3_-N), nitrite (NO_2_-N), total nitrogen (TN), and total phosphorous (TP) levels of the seawater samples were analyzed according to standard methods described by AQSIQ^57^.

### Samples preprocessing and elution of epiphytic bacteria

The elution of epiphytic bacteria from *G. lemaneiformis* followed the methods described by Burke^58^. Briefly, before extraction, algae samples were washed three times with filter-sterilized seawater to remove loosely associated microorganisms, microalgae, and phytoplankton. Five grams wet weight of *G. lemaneiformis* were placed into 250 mL conical flask containing 100 mL of calcium- and magnesium-free artificial seawater (CMFSW, 0.45 M NaCl, 10 mM KCl, 7 mM Na_2_SO_4_, 0.5 mM NaHCO_3,_ and 10 mM EDTA) and 1 mL filter-sterilized rapid multienzyme cleaner (3 M, Shanghai, China). Samples were incubated in an oscillating concentrator (DHZB-500, Jintan Science Analysis Instrument Co., Ltd.) for 3 h at 25 °C and 180 rpm. To completely elute the surface epiphytic bacteria on *G. lemaneiformis*, samples were sonicated (JY92-IIN, Ningbo Scientz Biotechnology Co., Ltd.) for 10 min (50 W, 5 s/5 s)^59^. Algal material was removed by sterilized tweezers and the remaining solution centrifuged at 8000 rpm for 20 min. The supernatant was removed from the upper layer, and about 1.0 mL of liquid was left at the bottom of the centrifuge tube to be mixed with the bacterial precipitate and transferred to new centrifuge tube before being centrifuged at 12,000 rpm for 5 min to completely remove the supernatant. The obtained bacterial precipitate was thoroughly washed once with 1 × TE buffer, followed by dissolving the bacterial precipitates in 1 mL 1 × TE buffer and stored at – 20 °C for DNA extraction.

### Detailed DNA extraction

The DNA from epiphytic bacteria on *G. lemaneiformis* was extraction by the procedure described previously^60^. Briefly, bacterial precipitate samples were centrifuged at 12,000 rpm for 5 min to remove the supernatant. Next, 100 μL TE buffer was added to each centrifuge tube to thoroughly suspended the bacterial precipitate, followed by the addition of 60 μL 10% SDS solution and 10 μl 20 mg·ml^−1^ proteinase K, and then mixed gently before being incubated at 37 °C for 1 h. After incubation, 100 μl 5 mol·L^−1^ NaCl solution was added to each sample and the tubes inverted repeatedly at least 10 times to mix, after which 80 μl CTAB/NaCl solution was added to each tube followed by gentle mixing and incubation at 65°C for 10 min. Next, 700 μl Chloroform: Isoamyl Alcohol (24:1) was added and the samples mixed gently before being centrifuged at 12,000 rpm for 2 min. The supernatant was collected and transferred to new sterile 1.5 mL polypropylene centrifuge tubes, followed by the addition of an equal volume of Phenol: Chloroform: Isoamyl Alcohol (25:24:1), mixed gently and centrifuged at 12,000 rpm for 2 min. The supernatant was transferred to new sterile 1.5 mL Eppendorf EP tubes. DNA was precipitated by mixing the supernatant with 2X cold absolute ethanol, and the precipitated DNA collected by centrifugation at 12,000 rpm and 4°C for 15 min. The pelleted DNA was washed with 300 μl of 70% cold ethanol and centrifuged at 12,000 rpm and 4°C for 15 min. Residual supernatant was removed by snap centrifugation followed by drying DNA pellets for 5–10 min on a clean bench. Next, DNA was resuspended in 100 μl TE buffer solution and the DNA concentration and purity measured using a NanoDrop ND-1000 photometer (Thermo Scientific). DNA integrity was checked on Agarose Gel Electrophoresis and stored at – 20 °C.

### PCR amplification of the 16S rRNA gene and sequencing

The amplification of the 16S rRNA gene was carried out using the primers (16S rRNA V3-V4 341F: 5′-CCTAYGGGRBGCASCAG-3′ and 16S rRNA V3-V4 806R: 5′-GGACTACNNGGGTATCTAAT-3′) with a standard PCR protocol^61^. The PCR amplification program was performed as following: 95 °C for 5 min, 27 cycles, where 1 cycle consisted of 95 °C for 30 s (denaturation), 55 °C for 30 s (annealing), 72°C for 45 s (extension), and a final extension at 72 °C for 7 min. The quality of PCR products was verified by 1% agarose electrophoresis gel. PCR products were purified with a PCR fragment purification kit (TaKaRa Biotech, Japan). The Illumina high-throughput sequencing (Hiseq 2500 PE250) technique was used to study the diversity of epiphytic bacteria associated with *G. lemaneiformis*. Sequencing and bioinformatic analysis were performed at Total Genomics Solution (, Shenzhen, China). The sequence data reported in this study have been deposited in the NCBI GenBank database under the accession number SRP198594. All SRA data are available at: https://www.ncbi.nlm.nih.gov/sra/PRJNA543182.

### Denoising of sequence data

Perl script was used to split the offline data into different sample data according to the barcode sequence, while the barcode and PCR primer sequences were cut off^62^. Splicing of PE Reads followed the following steps^63^: (1) Bases below the mass value of 20 at the end of the read were filtered and a 50 bp window was set. When the average mass value in the window was less than 20, the back-end base is cut off from the window and the read below 50 bp after quality control are filtered. (2) According to the overlapping relationship between PE reads, pair of reads are merged to form a sequence, with a minimum overlap length of 10 bp. (3) The allowable maximum mismatch ratio of the overlap region of the splice sequence was 0.2 and non-conforming sequences were screened. (4) Samples were distinguished according to the barcode at both ends of the sequence and the primer, and the sequence direction was adjusted. The mismatch number allowed by the barcode was 0, and the maximum primer mismatch number was 2. The Tags sequences obtained after the above processing are compared with the database (Gold database, http://drive5.com/uchime/uchime_download.html) to detect chimera sequences, and finally, the chimera sequences were removed to obtain the final Effective Tags^64^.

### OTU and species communities analysis

The Uparse software (Uparse v7.0.1001, http://drive5.com/uparse/) was used to perform 97% identify clustering of all Effective Tags sequences of all samples to form OTU^65^. The representative OTU sequences were selected and compared with GreenGene (16S chloroplast, mitochondria, http://greengenes.secondgenome.com/), RDP (16S, http://rdp.cme.msu.edu/), Silva (18S, http://www.arb-silva.de) and Unite (ITS, http://unite.ut.ee/index.php) database using the assign_taxonomy.py (http://qiime.org/scripts/assign_taxonomy.html) script and RDP Classifier method^66^ in Qiime to obtain species annotation information (the default confidence threshold was above 0.8). OTU and its Tags annotated as chloroplast or mitochondria and not annotated to the boundary level were removed. Perl script was used for statistics of Effective Tags data of each sample, low-frequency Tags data, Tags annotation data, data annotated by chloroplast and mitochondria, and the number of OTU obtained for each sample^67^. The R software was also used to carry out statistical analysis of the annotation proportion of each classification level of OTU and the relative abundance of species in each classification level. The relative abundance heatmaps of OTU level and genus level, and cluster analysis between samples and species were performed R software.

### Alpha and beta diversity analysis

Alpha_diversity.py (http://qiime.org/scripts/alpha_diversity.html) script in the QIIME software package was used to calculate four diversity indexes (Observed species, Chao, Shannon, phylogenetic diversity)^68^ based on the uniform OTU abundance table. The calculation of phylogenetic diversity required the phylogenetic tree data of OTU. Jackknifed_beta_diversity.py (http://qiime.org/scripts/jackknifed_beta_diversity.html) script in the QIIME software package was used to calculate three beta diversity distances (Bray Curtis, Weighted Unifrac, Unweighted Unifrac) based on the homogeneous OTU abundance table^69^. For nMDS, R software was used for nMDS analysis, and the drawing was based on the uniform OTU abundance table^70^.

### Correlation analysis of environmental factors

For redundancy analysis (RDA), the RDA function in the vegan package was used for ranking analysis^47^. Through envfit function, r^2^ and P values of the influence of each environmental factor on species distribution was calculated, followed by RDA analysis to screen out environmental factors with significant influence. Applying the ‘bioenv’ function in the vegan package, the environmental factor or combination with the largest correlation with the species matrix (Spearman) were screened out, and the environmental factor obtained by screening was analyzed by targeted RDA. For Spearman correlation, Spearman correlation values of species and environmental factors were calculated using the ‘Corr. test’ function of psych package in R and its significance tested^30^. The pheatmap function of the pheatmap package was used for visualization. The vegan package in R was used for the Mantel test^71^. Vegdist function was used to transform the distance matrix for ‘species matrix’ and ‘environmental factor data matrix’. The Spearman correlation analysis was performed for the two types of matrices with the Mantel function to obtain r and P values.

### Functional annotation analysis

PICRUSt (http://huttenhower.sph.harvard.edu/galaxy/root?tool_id=PICRUSt_normalize) was used to predict the function of microbial communities^72^ based on 16S rDNA sequencing. The functional abundance table of each level was obtained. R software was used to draw the heatmap of the functional abundance spectrum, and also for species, functional consistency analysis, and mapping.

### Statistical analysis of data

The physicochemical factors of seawater and the alpha-diversity of epiphytic bacterial communities were analyzed using one-way ANOVA. The analysis of significant difference of data was carried out using SPSS 19.0 software, with the significant threshold set to 0.05. The difference among the EBC at the three geographic locations was performed by the Permutational Multivariate Analysis of Variance (PERMANOVA) with Adonis function from the vegan package in R software^30^.

## Supplementary Information

Supplementary Information.

## Data Availability

The datasets presented in this study can be found in online repositories, the names of the repository/repositories and accession number(s) can be found in the article/Supplementary Material.
