# Author Correction: Environmental factors shape the epiphytic bacterial communities of *Gracilariopsis lemaneiformis*

**DOI:** 10.1038/s41598-022-17516-1

**Published:** 2022-08-02

**Authors:** Pengbing Pei, Muhammad Aslam, Hong Du, Honghao Liang, Hui Wang, Xiaojuan Liu, Weizhou Chen

**Affiliations:** 1 Institute of Marine Sciences, Guangdong Provincial Key Laboratory of Marine Biotechnology and STU‑UNIVPM Joint Algal Research Center, College of Science, Shantou University, Shantou, China; 2 Southern Marine Science and Engineering Guangdong Laboratory (Guangzhou), Guangzhou, China; 3 Faculty of Marine Sciences, LUAWMS, Lasbela, Pakistan

Correction to: *Scientific Reports* https://doi.org/10.1038/s41598-021-87977-3, published online 21 April 2021

The original version of this Article contained an error in Affiliation 2, which was incorrectly given as ‘Southern Marine Science and Engineering Guangdong Laboratory, Guangzhou, China’. The correct affiliation is listed below:

Southern Marine Science and Engineering Guangdong Laboratory (Guangzhou), Guangzhou, China

The original Article has been corrected.

