# Supplementary material for: Environmental factors shape the epiphytic bacterial communities of Gracilariopsis lemaneiformis
Source: Sci Rep. 2021 Apr 21;11:8671. doi: 10.1038/s41598-021-87977-3 (PMC8060329; doi:10.1038/s41598-021-87977-3)
Supplement: Supplementary file 1 — Supplementary Information. [file 41598_2021_87977_MOESM1_ESM.doc]

# Environmental Factors Shape the Epiphytic Bacterial Communities of *Gracilariopsis lemaneiformis*

Pengbing Pei^1,2^, Muhammad Aslam^1,2,3*^, Hong Du^1,2,*^, Honghao Liang^1,2^, Hui Wang^1,2^, Xiaojuan Liu^1,2^, Weizhou Chen^1,2^

^1^Institute of Marine Sciences, Guangdong Provincial Key Laboratory of Marine Biotechnology and STU-UNIVPM Joint Algal Research Center, College of Science, Shantou University, Shantou, China

^2^Southern Marine Science and Engineering Guangdong Laboratory (Guangzhou), Guangzhou, China

3 Faculty of Marine Sciences, LUAWMS-Pakistan.

*** Correspondence:** H Du, [hdu@stu.edu.cn and M. Aslam, drmaslam@hotmail.com;](mailto:hdu@stu.edu.cn and M. Aslam, drmaslam@hotmail.com; %0C)

[***Supplementary Material***](mailto:hdu@stu.edu.cn and M. Aslam, drmaslam@hotmail.com; %0C)

**Supplementary Table S1.** Average (±SD of nine replicates) value of environmental factors in seawater surrounding the *G. lemaneiformis* from three locations. Different letters (a, b, c) denote significant (*p* < 0.05) differences in mean value between NR, NA, and LJ in seawater samples.

| **Environmental factors** | **NR** | **NA** | **LJ** |
| --- | --- | --- | --- |
| Temp (℃) | 13.713±0.178^a^ | 13.872±0.288^a^ | 12.214±0.248^b^ |
| pH | 7.951±0.012^ab^ | 7.829±0.155^a^ | 8.031±0.014^b^ |
| Sal (ppt) | 30.919±1.503^a^ | 30.510±0.711^ab^ | 28.959±0.962^b^ |
| DO (mg·L^-1^) | 8.156±0.115^a^ | 8.001±0.073^a^ | 8.923±0.021^b^ |
| EC (×10^3^ μs·cm^-1^) | 37.223±1.938^a^ | 36.218±1.478^ab^ | 33.529±0.903^b^ |
| TDS (ppm) | 30.829±1.513^a^ | 30.406±0.548^a^ | 29.150±0.937^a^ |
| NH_4_-N (mg·L^-1^) | 0.016±0.002^a^ | 0.018±0.003^a^ | 0.006±0.002^b^ |
| NO_3_-N (mg·L^-1^) | 0.384±0.025^a^ | 0.293±0.015^b^ | 0.325±0.022^b^ |
| NO_2_-N (mg·L^-1^) | 0.002±0.0006^a^ | 0.004±0.0001^a^ | 0.011±0.0005^b^ |
| DIN (mg·L^-1^) | 0.401±0.025^a^ | 0.316±0.019^b^ | 0.341±0.023^b^ |
| TN (mg·L^-1^) | 3.351±0.070^a^ | 3.055±0.054^b^ | 2.668±0.047^c^ |
| TP (mg·L^-1^) | 0.088±0.006^a^ | 0.069±0.005^b^ | 0.055±0.006^b^ |

**Supplementary Table S2.** Shannon index, Chao1 index, Observed_species index and PD_whole_tree index showed the α-diversity of EBC on *G. lemaneiformis* at NR, NA, and LJ. Different letters (a, b) denote significant (*p* < 0.05) differences in mean value between NR, NA, and LJ in *G. lemaneiformis* samples.

| **Alpha-diversity** | **NR** | **NA** | **LJ** |
| --- | --- | --- | --- |
| Shannon index | 5.482±0.654^a^ | 4.832±0.184^a^ | 5.105±0.652^a^ |
| Chao1 index | 303.612±37.046^a^ | 238.612±11.656^b^ | 270.216±28.405^ab^ |
| Observed_species index | 268.389±25.716^a^ | 195.444±8.878^b^ | 228.222±12.075^b^ |
| Phylogenetic diversity index | 18.708±1.268^a^ | 14.544±0.792^b^ | 15.897±1.126^b^ |

**Supplementary Figure S1.** The EBC composition at family level on *G. lemaneiformis* at NR, NA, and LJ. Species richness represented less than 1% of the total bacteria in all samples were grouped into Rare taxa.

**Supplementary Table S3.** Affiliations of EBC composition in *G. lemaneiformis* samples at family level. Different letters (a, b) denote significant (*p* < 0.05) differences in mean value between NR, NA, and LJ in *G. lemaneiformis* samples.

| Taxonomy | Relative abundance /% | | | Affiliation |
| --- | --- | --- | --- | --- |
|  | NR | NA | LJ |  |
| Flavobacteriaceae | 32.911^a^ | 22.584^a^ | 14.310^a^ | Bacteroidetes |
| Cellvibrionaceae | 2.331^a^ | 18.616^ab^ | 31.733^b^ | Gammaproteobacteria |
| Saprospiraceae | 3.670^a^ | 6.423^ab^ | 10.334^b^ | Bacteroidetes |
| Hyphomonadaceae | 6.982^a^ | 8.718^a^ | 4.384^a^ | Alphaproteobacteria |
| Thiohalorhabdaceae | 1.265^a^ | 5.048^b^ | 4.018^ab^ | Gammaproteobacteria |
| Ruminococcaceae | 10.250^a^ | 0.325^b^ | 0.116^b^ | Firmicutes |
| Sphingomonadaceae | 2.852^ab^ | 0.990^a^ | 3.887^b^ | Alphaproteobacteria |
| Microtrichaceae | 2.070^a^ | 0.515^a^ | 5.088^a^ | Actinobacteria |
| Rhodobacteraceae | 0.989^a^ | 1.030^a^ | 4.156^b^ | Alphaproteobacteria |
| Trueperaceae | 1.837^a^ | 5.650^b^ | 1.158^a^ | Deinococcus-Thermus |
| Nitrincolaceae | 0.061^a^ | 6.692^a^ | 0.711^a^ | Gammaproteobacteria |
| Thiotrichaceae | 0.303^a^ | 5.044^b^ | 1.178^a^ | Gammaproteobacteria |
| Muribaculaceae | 6.293^a^ | 0.012^b^ | 0.010^b^ | Bacteroidetes |
| Lachnospiraceae | 5.503^a^ | 0.503^b^ | 0.202^b^ | Firmicutes |
| Halomonadaceae | 6.468^a^ | 0.058^a^ | 0.002^a^ | Gammaproteobacteria |
| Rare taxa | 5.120^a^ | 3.255^a^ | 3.570^a^ |  |

**
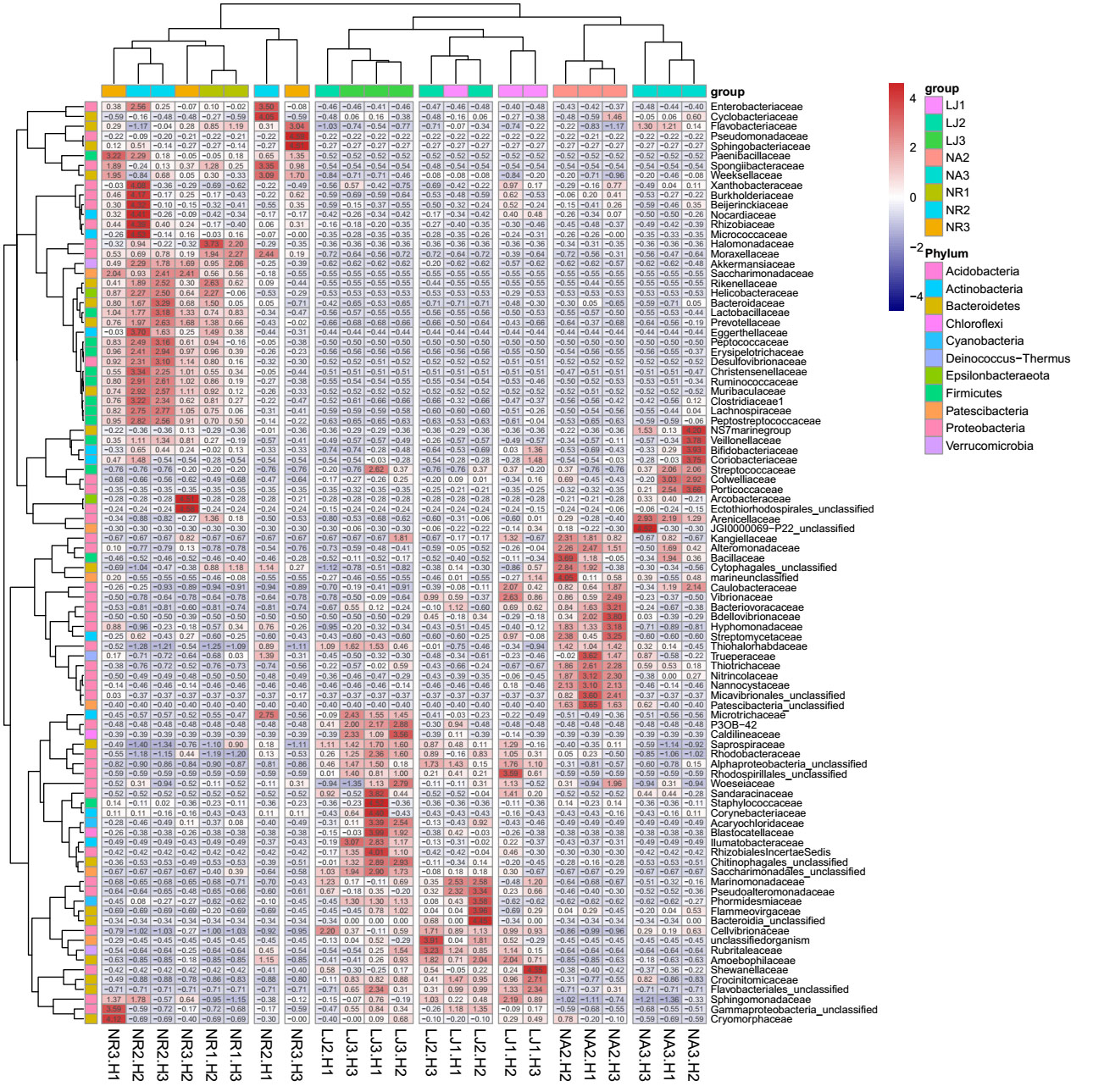
**

**Supplementary Figure S2.** Family level distribution of EBC on *G. lemaneiformis* at three different geographic locations.

**Supplementary Table S4.** Percentage of metagenomic sequences of functional composition of Level 1 of the EBC on *G. lemaneiformis*.

| Level 1 | Level 2 | Relative abundance /% | | |
| --- | --- | --- | --- | --- |
|  |  | NR | NA | LJ |
| Metabolism | Amino Acid Metabolism | 10.039 | 11.037 | 11.104 |
|  | Biosynthesis of Other Secondary Metabolites | 1.002 | 1.027 | 1.033 |
|  | Carbohydrate Metabolism | 9.586 | 9.555 | 9.864 |
|  | Energy Metabolism | 7.175 | 6.477 | 7.011 |
|  | Enzyme Families | 2.238 | 2.012 | 2.091 |
|  | Glycan Biosynthesis and Metabolism | 2.063 | 2.095 | 1.864 |
|  | Lipid Metabolism | 3.130 | 3.541 | 3.639 |
|  | Metabolism of Cofactors and Vitamins | 4.942 | 4.805 | 4.936 |
|  | Metabolism of Other Amino Acids | 1.796 | 2.098 | 2.030 |
|  | Metabolism of Terpenoids and Polyketides | 2.052 | 2.272 | 2.396 |
|  | Nucleotide Metabolism | 3.516 | 3.402 | 3.430 |
|  | Xenobiotics Biodegradation and Metabolism | 2.615 | 3.167 | 3.421 |
| Genetic Information Processing | Folding, Sorting and Degradation | 2.532 | 2.540 | 2.501 |
|  | Replication and Repair | 7.693 | 7.281 | 7.240 |
|  | Transcription | 2.350 | 2.280 | 2.227 |
|  | Translation | 4.837 | 4.694 | 4.545 |
| Cellular Processes | Cell Growth and Death | 0.629 | 0.766 | 0.684 |
|  | Cell Motility | 2.266 | 2.808 | 2.115 |
| Environmental Information Processing | Membrane Transport | 10.834 | 8.556 | 9.390 |
|  | Signal Transduction | 1.947 | 2.043 | 1.899 |
| Human Diseases | Infectious Diseases | 0.489 | 0.474 | 0.479 |
| Unclassified | Cellular Processes and Signaling | 3.684 | 3.881 | 3.469 |
|  | Genetic Information Processing | 2.794 | 2.766 | 2.594 |
|  | Metabolism | 2.776 | 2.879 | 2.697 |
|  | Poorly Characterized | 5.198 | 5.405 | 5.203 |
| Others | Others | 1.820 | 2.140 | 2.138 |

**Supplementary Figure S3.** Percentage of metagenomic sequences of functional composition of Level 1 of the EBC on *G. lemaneiformis* at NR, NA, and LJ.

**Supplementary Figure S4.** Percentage of metagenomic sequences of functional composition of Level 3—Amino acid metabolism—of the EBC on *G. lemaneiformis* at NR, NA, and LJ.

**Supplementary Figure S5.** Percentage of metagenomic sequences of functional composition of Level 3—Carbohydrate metabolism—of the EBC on *G. lemaneiformis* at NR, NA, and LJ.

**Supplementary Figure S6.** Percentage of metagenomic sequences of functional composition of Level 3—Energy metabolism—of the EBC on *G. lemaneiformis* at NR, NA, and LJ.

**Supplementary Table S5.** The EBC samples of *G. lemaneiformis* used in present study. Raw PE: the raw paired-end (PE) reads. Effective tags: the number of tags sequences that will eventually be used for subsequent analysis after filtering the chimera. Effective (%): the percentage between the number of effective tags and the number of PE reads in raw data. ‘—’ represent data missing due to experimental reason.

| **Location** | **Group name** | **Sample name** | **Date** | **Raw PE (#)** | **Effective Tags (#)** | **Effective (%)** | **OTUs** |
| --- | --- | --- | --- | --- | --- | --- | --- |
| Nan’ao Island  (NA) | NA2 | **NA2_1** | 03/02/2018 | 44603 | 43670 | 97.91 | 498 |
|  |  | **NA2_2** | 03/02/2018 | 38852 | 37380 | 96.21 | 592 |
|  |  | **NA2_3** | 03/02/2018 | 54001 | 52713 | 97.61 | 573 |
|  | NA3 | **NA3_1** | 03/02/2018 | 51036 | 48794 | 95.61 | 352 |
|  |  | **NA3_2** | 03/02/2018 | 47343 | 46242 | 97.67 | 498 |
|  |  | **NA3_3** | 03/02/2018 | 43518 | 42484 | 97.62 | 384 |
| Nanri Island  (NR) | NR1 | **NR1_1** | 11/01/2018 | — | — | — | — |
|  |  | **NR1_2** | 11/01/2018 | 53313 | 52517 | 98.51 | 744 |
|  |  | **NR1_3** | 11/01/2018 | 48339 | 47810 | 98.91 | 647 |
|  | NR2 | **NR2_1** | 11/01/2018 | 36789 | 36178 | 98.34 | 526 |
|  |  | **NR2_2** | 11/01/2018 | 49979 | 48986 | 98.01 | 696 |
|  |  | **NR2_3** | 11/01/2018 | 37501 | 36734 | 97.95 | 658 |
|  | NR3 | **NR3_1** | 11/01/2018 | 52127 | 46398 | 89.01 | 786 |
|  |  | **NR3_2** | 11/01/2018 | 36247 | 35579 | 98.16 | 691 |
|  |  | **NR3_3** | 11/01/2018 | 44031 | 43313 | 98.37 | 562 |
| Lianjiang County  (LJ) | LJ1 | **LJ1_1** | 28/01/2018 | 47152 | 44042 | 93.4 | 540 |
|  |  | **LJ1_2** | 28/01/2018 | 47272 | 45685 | 96.64 | 593 |
|  |  | **LJ1_3** | 28/01/2018 | 58334 | 55715 | 95.51 | 540 |
|  | LJ2 | **LJ2_1** | 28/01/2018 | 40857 | 39974 | 97.84 | 583 |
|  |  | **LJ2_2** | 28/01/2018 | 46033 | 44472 | 96.61 | 591 |
|  |  | **LJ2_3** | 28/01/2018 | 41060 | 40089 | 97.64 | 444 |
|  | LJ3 | **LJ3_1** | 28/01/2018 | 58478 | 56311 | 96.29 | 544 |
|  |  | **LJ3_2** | 28/01/2018 | 52692 | 51732 | 98.18 | 489 |
|  |  | **LJ3_3** | 28/01/2018 | 56505 | 54517 | 96.48 | 629 |
